# Occupational accidents in Slovak Military Forests and Estates: incidence, timing, and trends over 10 years

**DOI:** 10.3389/fpubh.2024.1369948

**Published:** 2024-03-22

**Authors:** Michal Allman, Zuzana Dudáková, Martin Jankovský

**Affiliations:** Department of Forestry Technologies and Construction, Czech University of Life Sciences Prague, Prague, Czechia

**Keywords:** forestry, employees, occupational accidents, incidence rate, accident frequency, severity of accidents

## Abstract

**Introduction:**

Forestry provides a wide range of employment opportunities worldwide and is seen as one of the high-risk industries in terms of occupational accidents.

**Objectives:**

The submitted study analyzed the injury rate in the Military Forests and Estates of the Slovak Republic (62.6 thousand ha) between 2013 and 2022.

**Methods:**

The data analyses included regression and correlation analyses, χ^2^ tests to analyze the relationships between studied variables, and incidence rates.

**Results:**

During the observed period, employees suffered 26 occupational accidents, of which 19.2% were light, 57.7% were registered, 23.1% were severe, and 0% were fatal. For every 1 million m3 of harvested timber, 7.7 accidents occurred. The incidence rate during the observed period was 672.1/100,000 employees. The highest proportion of accidents was in the age group 51–60  years and in employees with the lowest length of work experience <5  years. Regarding time, the highest proportion of occupational accidents occurred between 8:01 and 10:00  AM (53.8%) and day-wise on Thursdays (46.2%). The highest proportion of accidents occurred among forest workers (65.3%) during pruning and silviculture activities (42.3%). The most common injury site was forest stands (65.3%). Superficial injuries (34.6%) were the most common, mainly affecting the lower limbs (50%). The most frequent material agents causing the accidents were work and transport areas as sources of worker fall (38.5%), and the most frequent reason for an accident to occur was the lack of personal requirements for proper work performance (92.4%), whereas only (3.8%) of accidents occurred due to the use of forbidden or hazardous working procedures.

**Conclusion:**

The presented study identified the most vulnerable worker groups and provided an overview of the overall injury rate at the state forest company in Slovakia. The documentation can be incorporated into the safety strategies of forest enterprises.

## 1 Introduction

Forests cover 4.06 billion ha (31%) of the world’s land surface (1) and provide several irreplaceable, non-productive services (2, 3). They are a place of rest, relaxation, and sports activities (4, 5). However, their great productive importance cannot be overlooked, and they provide numerous goods to human societies (3, 6). Forests provide 3.797 trillion m^3^ of timber (7) as a renewable natural resource with a wide range of uses (8, 9). Being an essential bioeconomy sector, forestry employs a large number of people in the world (10).

Approximately 33 million people (1% of global employment) are estimated to work directly in the formal and informal forest-based industries (1, 11, 12), of which 19.4 million produce lumber and wooden products and 8.06 million in forestry and forest harvesting (12). Several authors (13–16) report that forest-based industries are at high risk regarding accident rates. Feyer et al. (17) note that the injury rate of forest workers in New Zealand is four times higher than that of other occupational groups, with a mean annual 121 deaths per 100,000 workers (16). The fatal accident rate in the United States forestry was 19 times higher than in other sectors (13), whereas in Australia, forestry workers suffer two to three times more accidents than workers in other industries (18). A comparison of fatal occupational accidents in the agriculture, forestry, and fisheries sector between the EU (349) and the United States (211) is provided in its study (19). Since forestry is assigned to agriculture and fisheries, it is impossible to determine exactly how many accidents occur in the forestry sector. However, according to the ILO, forest workers were involved in accidents three to four times more frequently than agricultural workers (15). According to Eurostat’s 2021 injury database, within the EU, the highest proportion of fatal and non-fatal accidents is in the Forestry and Forestry logging sector in Germany (3,288 accidents; 51,300 employees on total), followed by Spain (2,735 accidents; 27,200 employees on total) and Italy (1,279 accidents; 41,700 employees on total). Romania had the highest number of fatal accidents in forestry at 18 (49,900 employees on total), followed by Germany with 10 and Spain with 8 (20). Additionally, Cho et al. (21) reported that in Korean forestry, which employed between 77,000 and 101,000 workers in the years 2010–2020, 1,543 occupational accidents occurred annually, of which 20 were fatal.

There are several reasons for the high accident rate in forestry. Laschi et al. (22) list the most significant factors: (i) terrain conditions; (ii) weather; (iii) biological agents (iv) use of machines and tools; (v) exposure to heavy loads; (vi) exposure to noise, vibration, wood dust, exhaust gasses, and other physical agents. FAO et al. (10) similarly state that work in forests is associated with inherently high occupational safety and health risks, primarily related to the use of heavy machinery, falling trees, climatic hazards, noise, vibration, non-ergonomic working postures, stress and strain, as well as exposure to various chemical and biological agents. Indeed, most authors attribute the risks in forestry mainly to the demanding working environment that contributes to specific risk factors acting on the workers and, subsequently, the high probability of an occupational accident (16, 17, 23–25).

To improve accident prevention measures, safety officers must first have detailed information on the accidents that already occurred, including their causes, the agents that caused them, and the employees who suffered the accidents. However, countries often do not keep a detailed accident registry, or workers, especially independent contractors, are not incentivized to report accidents. According to FAO et al. (10), the lack of reliable and comparable statistical data and research on occupational accidents and diseases persists, even though there are initiatives to document and analyze risks to the safety and health of forest workers. With this in mind, the main objective of this study is:

- to provide a detailed overview of the injury rate at the Military Forests and Estates of the Slovak Republic, SOE (MFE);
- to observe a potential relationship between the annual volume of harvested timber and the number of employees on occupational accident frequency, compare the accident frequency with the other state forest companies around the world;
- test the hypotheses that the age of the employees, their experience and temporal factors significantly affect occupational accident occurrence; and
- determine the activity, agents, modes of injuries, and location where the most occupational accidents occur.

## 2 Materials and methods

### 2.1 Information about the company, job descriptions, and employees in the company

**Figure 1:**
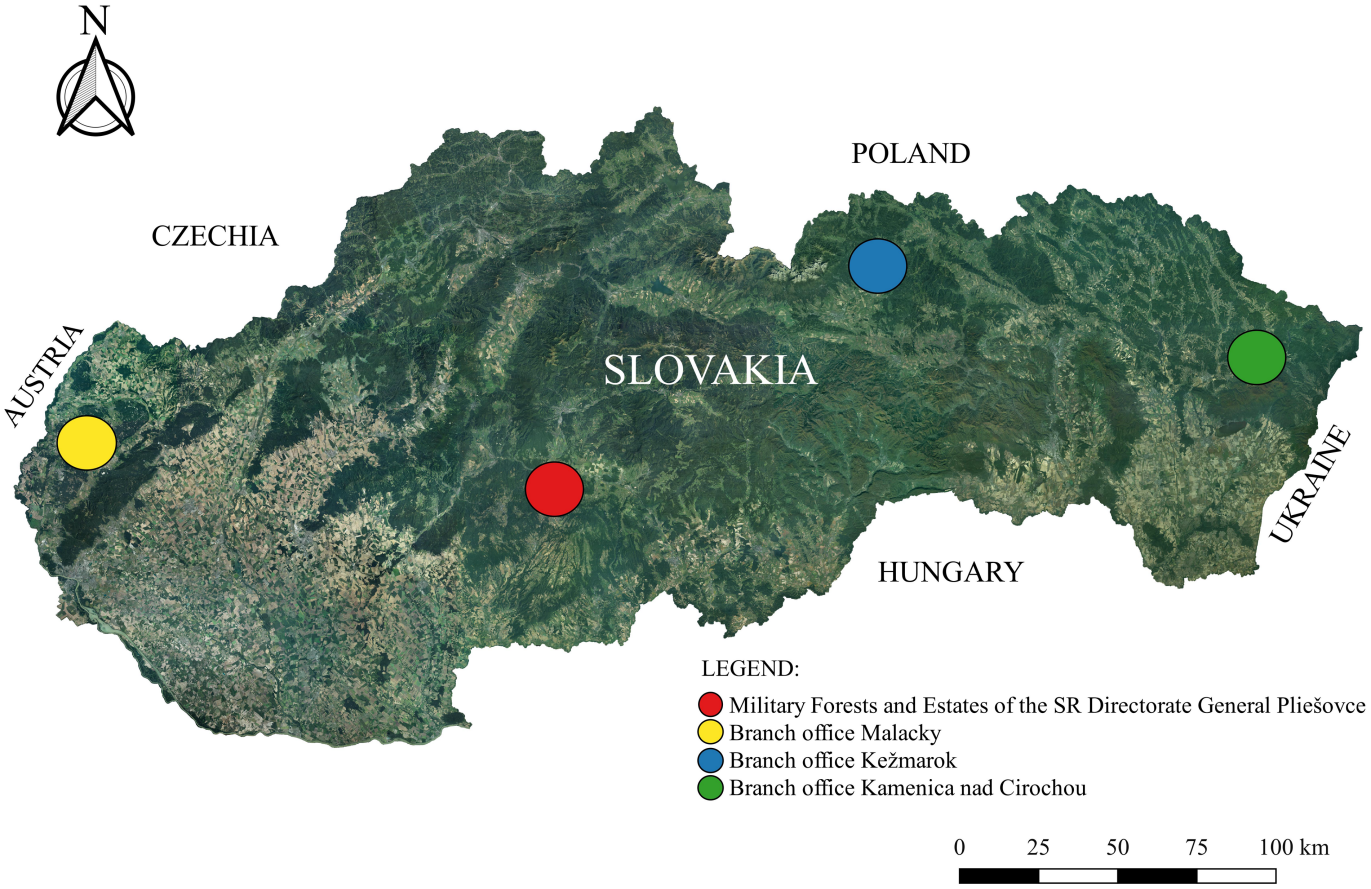
Location of individual organizational units of Military Forests and Estates in Slovakia.

The MFE was created to ecologically and efficiently manage the agricultural and forest land where the Slovak Army conducts military training. The company managed a total of 62,663 ha of forest land (6.2% of Slovak forests) with logging in 2022 at the level of 272,681 m^3^ (129,496 m^3^ deciduous and 143,185 m^3^ coniferous) (26). The most represented species were *Pinus sylvestris*—32.8%, *Fagus sylvatica*—29.7%, and *Picea abies*—16.3%. The company also farms on about 2,900 ha of agricultural land, mainly used for the production of feed for livestock and forest game and pastures for cattle grazing. Approximately 172 ha of agricultural land are used for short rotation poplar coppices. The enterprise consisted of a General Directorate located in Pliešovce (GPS: 48°25′33.09″ N; 19°9′11.33″E) and branch offices in Malacky (GPS: 48°26′14.55″N; 17°1′20.91″E), Kežmarok (GPS: 49°15′12.5″N; 20°31′47.98″E) and Kamenica nad Cirochou (GPS: 48°55′51.28″N; 22°0′3.35″E) (Figure 1).

The company employed office (38% of the staff) and manual workers. Office jobs included management of forest production, sales, and human resources. Foresters also partially work office jobs when implementing administrative activities. Field workers included workers in forest production and cultivation activities (pruning, cleaning, and culture protection), forest and agricultural activities, log yards, mechanics, and machine operators. In 2022, 6.2% of the staff worked outdoors primarily. The representation of men among employees was 75%.

Contractors supplied all harvesting conducted by the company. Considering harvesting volumes, the bulk of logging was conducted by the tree-length and whole-tree methods, using chainsaws (60%), followed by the cut-to-length (CTL) method, which was fully mechanized (40%). Extraction was carried out by skidders (57%), CTL technologies (40%), and cable yarders (3%).

Most of MFE’s employees work standard shifts from 7:00 AM to 3:00 PM with a lunch break of 30 min. The work weeks of most employees started on Mondays and ended on Fridays, except for drivers and mechanics, who sometimes worked irregular hours between 6:00 AM and after 4:00 PM. For work contractors, neither the working hours nor the scheduling during the working week were easily distinguished, as they organized their workdays independently.

### 2.2 The occupational safety and health system used

Accident records from the company were provided for 10 years (2012–2022) from the occupational accident records database at the Department of Safety and Health at Work of the company headquarters. Within the company, a certified safety officer supervised occupational safety and health (OSH) compliance, retrained employees in the field of OSH, and registered accidents. Since 2006, Act no. 500/2006 Coll. is in force (27), according to which the employer is obliged to keep records of serious and fatal occupational accidents for all employees, including work contractors. In the case of employees, a detailed, official accident report must be recorded if it is an accident where the employee lost 3 days of work, following Act no. 500/2006 Coll. Act no. 500/2006 transposed European Parliament Regulation No. 89/391/EEC into Slovak legislation (28) and the occupational accident report thus contains codes that follow the European Statistics on Accidents at Work (ESAW) (29).

### 2.3 Data and variables

Based on the information contained in the occupational accident reports and General Directorate of MFE, the following variables were identified and processed: (i) volume of harvested timber; (ii) number of employees; (iii) number of accidents at work; (iv) type of employment: employee, contractor; (v) sex: male, female; (vi) age (years); (vii) experience (years); (viii) the date on which the accident occurred; (ix) the time when the accident occurred; (x) a month of the year; (xi) job title; (xii) the activity in which the accident occurred; (xiii) the location of the accident; (xiv) severity of the accident at work: other—no days lost, registered—up to 3 days lost, severe—more than 3 days lost, excluding death, fatal; (xv) source of accident; (xvi) the cause of the accident; (xvii) type of injury; and (xvii) injured body-part.

### 2.4 Data and statistical analyses

Regression and correlation analysis was used to analyze the relationship between the volume of harvested timber and the number of employees, the volume of harvested timber, and the number of accidents at work. The incidence rate (29) (Eq. 1) was calculated as the number of accidents at work per 100,000 persons employed. It is a reliable metric that enables comparisons of occupational safety between companies, industries, or countries.


(1) $$\mathrm{Incidence}\;\mathrm{rate}\;\left(IR\right)=\frac{\mathrm{Number}\;\mathrm{of}\;\mathrm{accidents}\;\left(1012-2022\right)}{\begin{array}{c} \mathrm{Number}\;\mathrm{of}\;\mathrm{employed}\;\mathrm{persons}\\ \mathrm{in}\;MFE\;\left(2012-2022\right) \end{array}}\times 100\;000$$

The IR was observed over the long-term trend of the 10-year period from annual IRs. A chi-squared test was used to compare the significance of differences between variables. The null hypothesis was that there was no difference between the expected and observed data distribution. The test was used to compensate for differences in the development of the number of employees in the observed period, to compare the injury rate for individual years and further to the variables (vi), (vii), (viii), (ix), (x) (xi), (xii), and (xiii). Microsoft Excel and Statistica version 14.0.0.15 (Tibco, Software Inc.) were used to process and analyze the data.

## 3 Results

**Figure 2:**
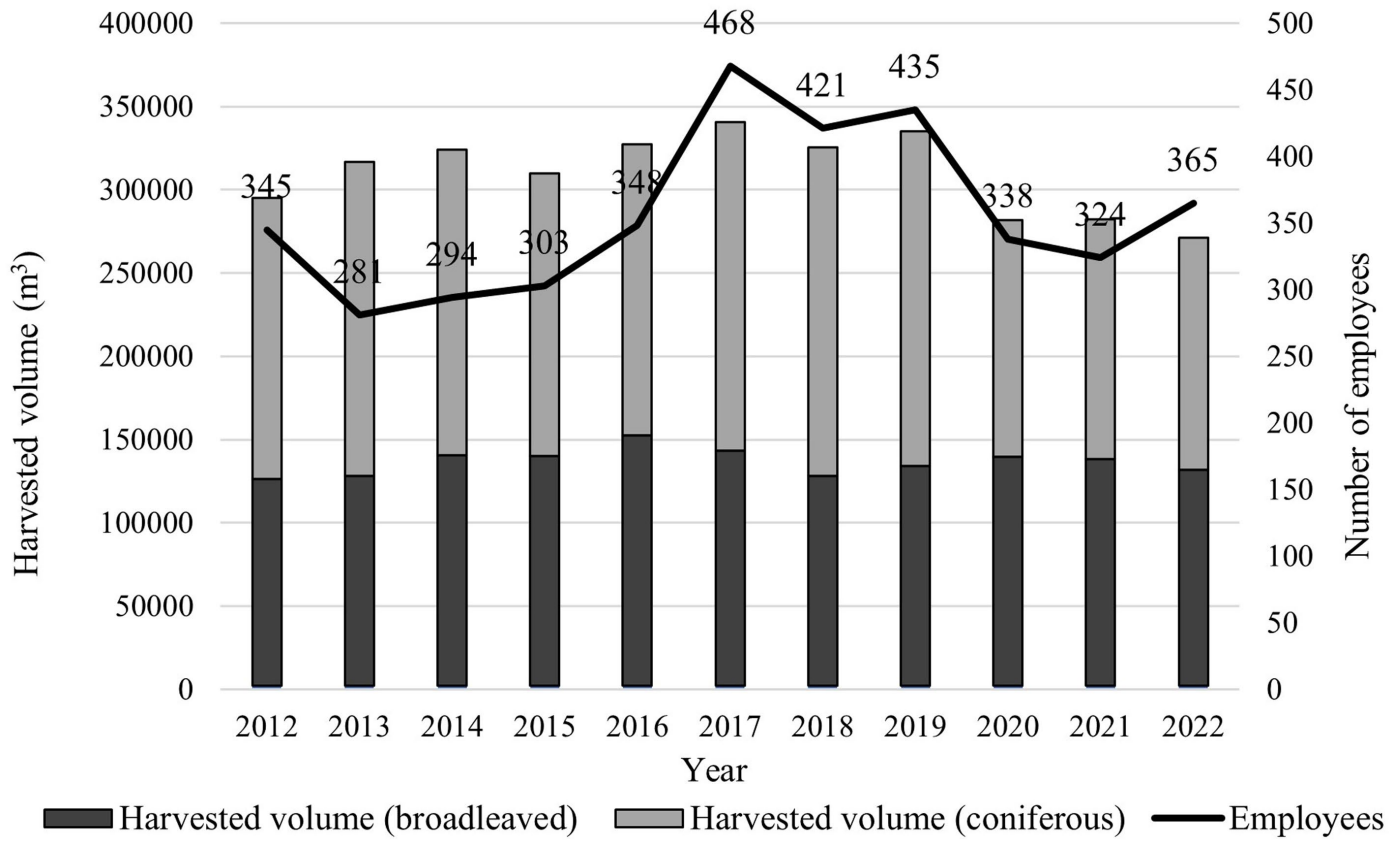
Volume of harvested timber and the development of the number of employees during the observed period.

The highest number of employees in the company was recorded in 2017 (Figure 2), namely 468. In the subsequent years, the number of employees decreased, along with harvesting volume, reaching the number 365 employees and approximately 270,000 m^3^ of harvested timber. The χ^2^ test confirmed that the number of employees within the observed period differed significantly between the particular years (103.5 > 18.31; df = 10; *p* = 0.00). A weak and insignificant relationship was observed between harvesting volume and the number of employees (*R* = 0.42, *R*^2^ = 0.18, *p* = 0.19), which was likely due to the fact that contractors carried out the harvesting.

**Figure 3:**
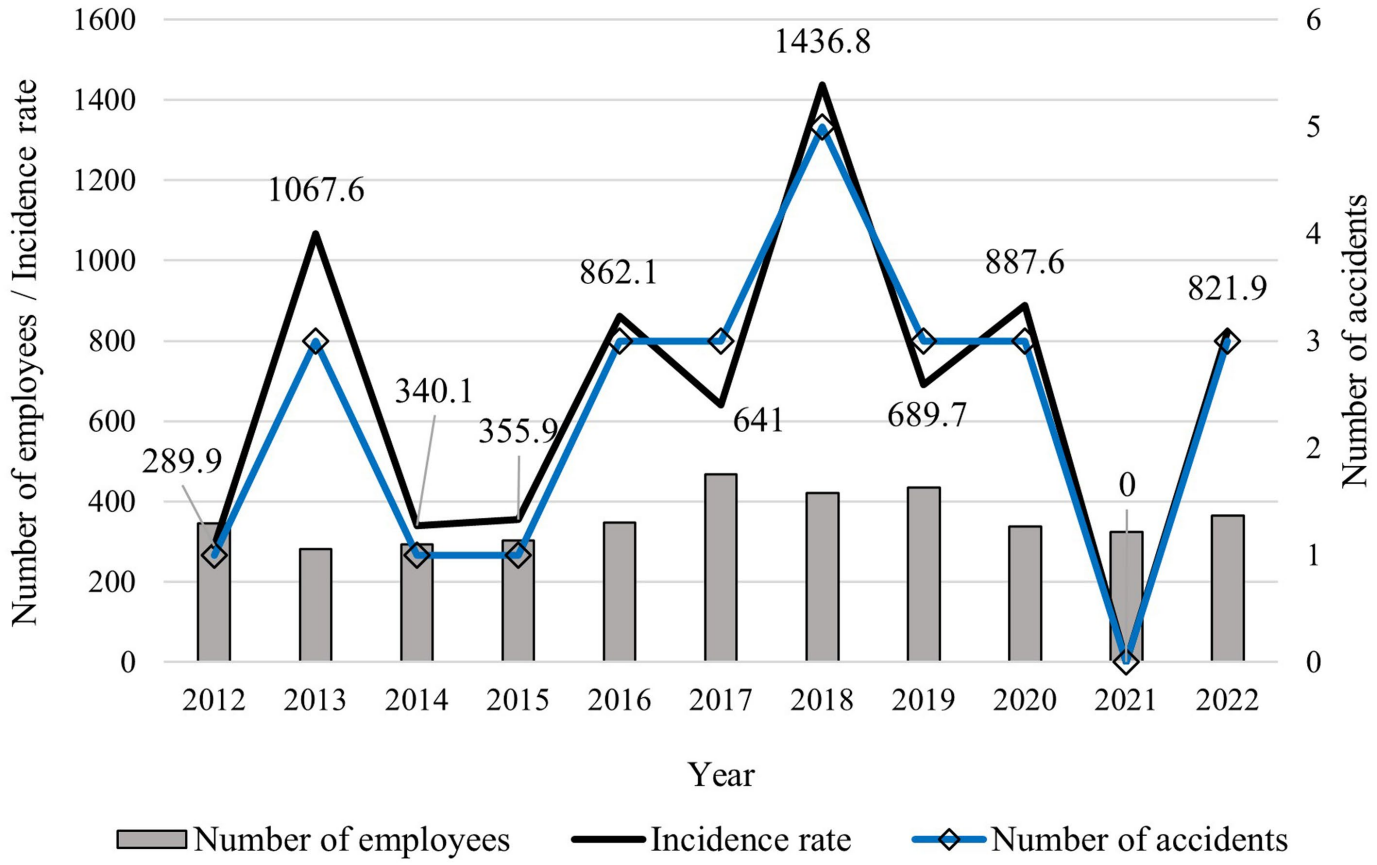
Number of employees and the development of the number of occupational accidents during the observed period.

During the observed period (2012–2022), 26 occupational accidents were suffered by company employees (Figure 3). The most injuries occurred in 2015 (5), the least in 2021, when no accident occurred. The χ^2^ test did not show significant differences in incidence rates from 1 year to another (87.05 < 18.31; df = 10; *p* = 0.506). Men suffered 85% of the occupational accidents, while women suffered 15%. Regarding accident severity, 19.2% were other, 57.7% were registered, 23.1% were severe, and 0% were fatal. Regression and correlation analysis between the harvesting volume and the number of occupational accidents showed a weak, insignificant relationship (*R* = 0.34, *R*^2^ = 0.11, *p* = 0.30). Recalculating the number of occupational accidents per million m^3^ of harvested timber showed a rate of 7.7 occupational accidents per million m^3^, consisting of 1.5 other, 4.4 registered, 1.8 severe, and 0 fatal accidents per 1 million m^3^ of harvested timber. The mean company IR for the observed period was 672.1 on average. Detailed development of the annual IRs can be seen in Figure 3.

**Figure 4:**
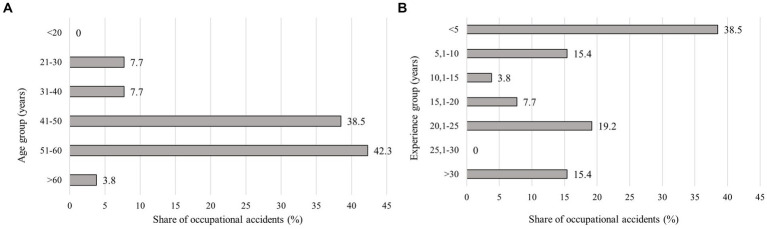
**(A)** Percentage of accidents by age group; **(B)** Percentage of accidents according to the employee experience.

The highest proportion of accidents was recorded in the age category 51–60 years, followed by 41–50 years. The lowest proportion of injuries was in the under 20-year-old category (Figure 4A). The χ^2^ test showed significant differences in accident frequency between the age groups (27.09 > 11.07; df = 5; *p* = 0.00). Considering the employee experience (Figure 4B), we can state that the highest frequencies were achieved by the least experienced employees, namely those with fewer than 5 years (38.5%) of experience. In contrast, employees with 25.1–30 years of experience suffered the fewest injuries (0). The χ^2^ test showed a significant difference in accident frequency between worker groups based on their experience (17.63 > 12.59; df = 6; *p* = 0.01).

**Figure 5:**
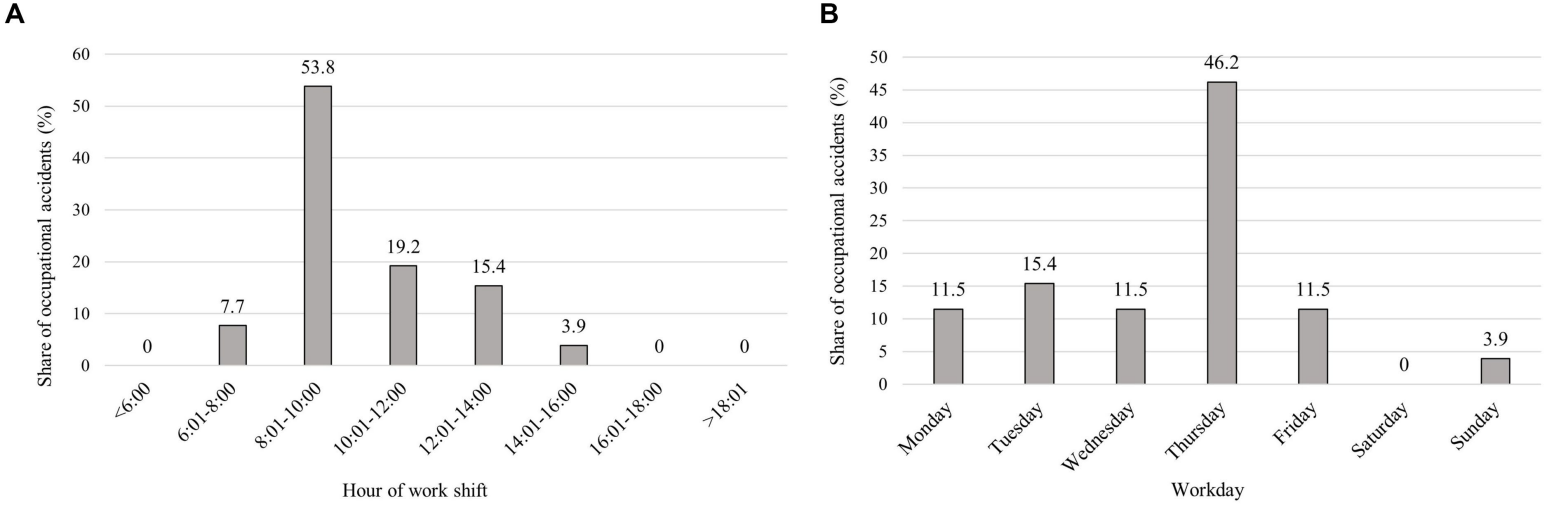
**(A)** Assessment of accident frequency within work shifts; **(B)** Percentage of accidents occurring by day of the week.

The percentage of accidents according to the time of their occurrence during the work shift can be seen in Figure 5A. The χ^2^ test confirmed a significant difference between the accident frequency during the work shift (48.46 > 14.07; df = 7; *p* = 0.00). The highest percentage of occupational accidents occurred between 8:01 AM and 10:00 AM, while the fewest occurred between 2:01 PM and 4:00 PM. Accident frequency within the work week (Figure 5B) showed that most accidents occurred on Thursday (46.2%). On the other days of the week, the distribution of frequencies was balanced, with the minimum on Sunday, when only one occupational accident was recorded and was connected to game management at the company hunting grounds. A significant difference in accident frequency between days of the week was also confirmed by the χ^2^ test (24.64 > 12.59; df = 6, 9 = 0.00). The monthly distribution of accidents during the year showed that the largest share of accidents (23.1%) occurred in August, followed by January, June, and October, each with 11.5% of accidents. No accidents happened in July and December. The χ^2^ test did not confirm a significant effect of the month of the year on accident frequency, thus, the differences were not statistically significant (12.80 < 19.68; df = 11; *p* = 0.306).

The breakdown of accident frequency by job and activity performed is provided by Table 1. The table shows that the largest share of accidents occurred among workers in forestry production and cultivation activities (65.3%), where workers prune trees by chainsaw or perform other cultivation activities with sharp tools directly in forest stands. In contrast, the lowest accident frequency was observed for clerks who worked in offices and processing line operators who worked in log yards. The χ^2^ test confirmed the existence of significant differences in accident frequency between jobs in the company (45.42 > 11.07; df = 5; *p* = 0.00), and we can conclude that field workers have the highest risk of occupational accidents occurring.

**Table 1:** Percentage of accidents by job, activity performed, and accident site.

Job	Accidents (%)	Activity performed	Accidents (%)	Accident site	Accidents (%)
Worker in forestry production	65.3	Pruning and cultivation activities	42.3	Forest stands	65.3
Forester, technician	11.5	Maintenance of buildings and equipment	23	Branch and district sites	19.2
Machine operator, mechanic	7.7	Vehicle ingress and egress	11.5	Workshops	7.7
Maintenance worker	7.7	Timber processing	7.7	Log yards	3.9
Processing line operator	3.9	Machine repairs	7.7	Quarries, sandpits	3.9
Office worker	3.9	Inspection of forestry workplaces	3.9	Office	0
		Hunting	3.9		

Following this, the effects of the activity performed on accident frequency were evaluated (Table 1). The most substantial proportion of occupational accidents occurred when pruning and cultivation activities were carried out (42.3%), followed by machine maintenance and repairs. The χ^2^ test confirmed a significant difference in accident frequency between the studied groups (21.41 > 12.59; df = 6; *p* = 0.00).

The most common site of accidents were forest stands (65.3%), where the workers were affected by a wide range of environmental factors (terrain, weather conditions), while no accident occurred in the office and indoors at individual branches during the observed period (Table 1). This difference in accident frequency based on site was confirmed as significant by the χ^2^ test (47.88 > 11.07; df = 5; *p* = 0.00).

**Figure 6:**
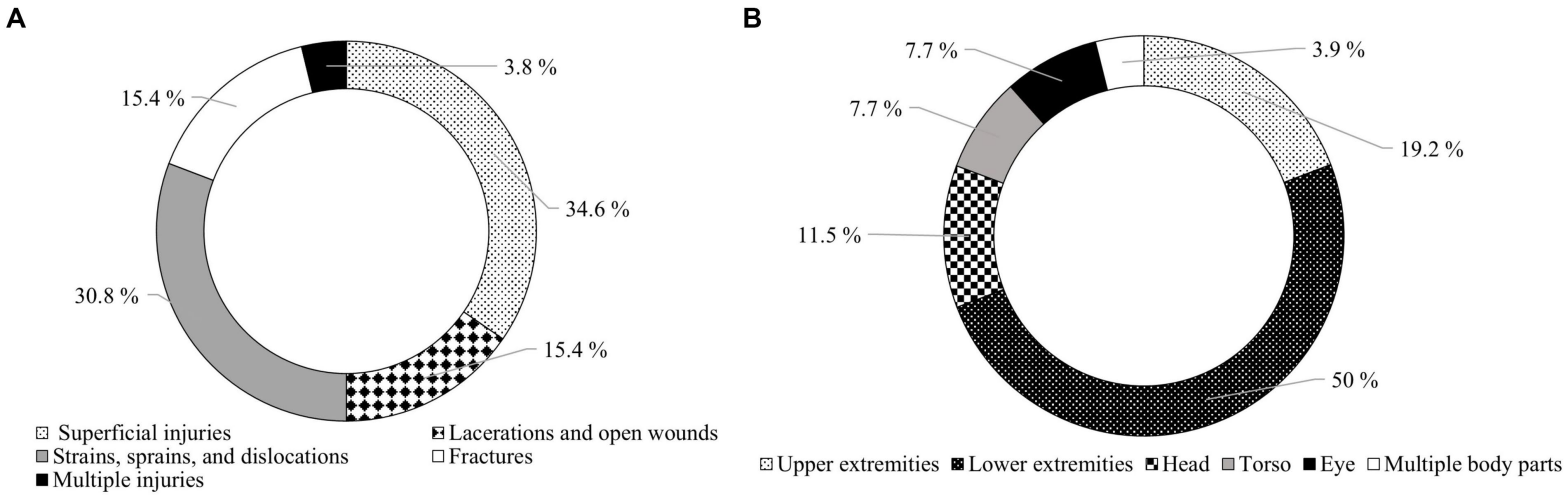
**(A)** Percentage of injury types; **(B)** Percentage of body parts injured.

The largest share of occupational accidents were superficial injuries (34.6%), followed by dislocations, sprains, and strains (30.8%), and multiple injuries (3.8%) had the smallest share (Figure 6A). The lower limbs (50%) were the most affected body parts, followed by the upper limbs (19.2%), while workers seldom suffered injuries to multiple body parts (3.9%) (Figure 6B).

Regarding the cause of occupational accidents, the most frequent cause (92.4% of accidents) was deficiencies in personal prerequisites for proper work performance (e.g., lack of physical abilities, sensory deficiencies, unfavorable personal characteristics, and immediate psychophysiological states). An equal 3.8% of accidents were due to (i) the faulty or unfavorable condition of the source of injury not the workplace and (ii) the use of hazardous procedures or methods of work, including acting without authorization, against orders, prohibitions, and instructions, or remaining in the endangered area. The χ^2^ test confirmed a statistically significant difference between the individual causes of accidents (14.77 > 5.99; df = 2; *p* = 0.00).

**Figure 7:**
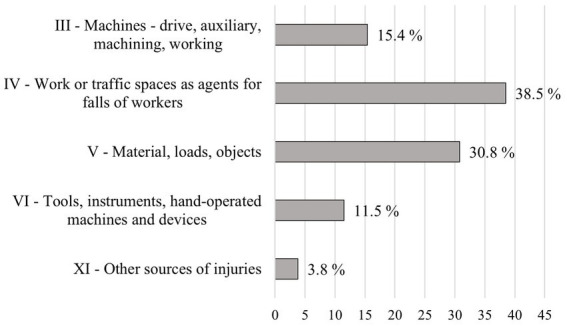
Percentage of occupational accidents by material agents.

An overview of the sources of occupational accidents in the company can be seen in Figure 7. The most common source of occupational accidents (38.5%) were workspaces as sources of falls, followed by materials, loads, and objects as sources of accidents (30.8%). The χ^2^ test confirmed a statistically significant difference between accident sources (10.53 > 9.49; df = 4; *p* = 0.003).

## 4 Discussion

The company OSH management’s effectiveness and enterprise data were compared with other countries. For instance, the MFE IR reached 672.1, a higher value than Akay et al. (30) reported as the mean IR for Turkish forestry (585.6), excluding fatal accidents. In Argentina, Peirano (31) reports 195 accidents per 1,000 forest harvesting workers. Compared to MFE, this is a higher number, though it was claimed only for harvesting workers. Further, Tsioras et al. (24) state an accident rate of 77.6 accidents per million cubic meters in Austria. At MFE, after converting the number of injuries to 1 million m^3^ of harvested timber, 7.7 occupational accidents occurred. Grzywiński et al. (32) state 11.14 occupational accidents per 1 million m^3^ of harvested timber in Poland. Compared to other countries, we can state that the company has lower accident frequency than those in Austria and Poland, mainly because of shifting the riskiest occupations to contractors. This fact reflects in the weak and insignificant relationship between the volume of harvested timber and the number of occupational accidents occurring in a given year, as well as Figure 3, where we can see that the number of employees had very little overlap with the trend in IR or number of accidents. The problem concerning the IR in these activities is that contractors do not report all accidents to the enterprise, as there is no legal obligation for it. They are only obliged to report severe and fatal accidents to the company according to act no. 311/2001 Coll. and Act no. 124/2006 Coll. Thus, the records on the accident rate in the private forestry sector did not document all accidents. Another reason for the lower accident frequency was the regular retraining of employees, demanding compliance with safe working procedures, and wearing personal protective equipment by both employees and contractors (regularly checked by safety officers at individual branches).

The presented study confirmed a higher injury rate among employees aged 41–50 and 51–60. In line with our study, Thelin (33), Wilhelmson et al. (34), Jankovský et al. (35), and Allman et al. (36) confirmed a higher injury rate in older age groups, while Thelin (33) and Wilhelmson et al. (34) also confirmed an increased injury rate among the youngest employees under 20, which we could not confirm for MFE. Feyer et al. (17) lists the oldest employees over 61 years as the most at-risk employee group. Laschi et al. (22) found the highest injury rate in the 31–50 years range, increasing with age. Slightly younger groups were confirmed to be the riskiest in studies by Bentley et al. (37) and Tsioras et al. (24) from the age of 30–45. On the contrary, while Salminen et al. (23) refuted the significance of the influence of age on injury rates.

Employee experience proved to be a significant factor influencing injury rates. The highest proportion of injuries (38.5%) occurred among employees with the shortest period of experience below 5 years, which was also reported by other authors (18, 35, 38–40). On the contrary, Errico et al. (41) notes that with increasing experience, workers begin to underestimate the risks at work and thus increase the accident rate, while Salminen et al. (23) found no relationship between experience and injury rate.

The time of occurrence of an accident at work during the work shift proved to be a significant factor, and most occupational accidents (53.8%) occurred between 8:01 and 10:00 AM. Several authors note increased injury rates just before lunch, between 10:00 AM and 12:00 PM (24, 35, 36). Multiple authors report a two-peak distribution throughout the workday—one before and one after lunch. The second peak injury rate during the day could be attributed to the lunch effect, according to which food consumption might be related to increased accident frequency in the hours following the lunch (24). In our case, the two-peak intraday injury distribution was not confirmed.

The highest proportion of work-related accidents within the week occurred on Thursday (46.2%). In other studies, Thursdays were confirmed as the days with the highest risk of occupational accidents occurring (18, 42). On the other hand, other authors report higher injury rates in the first half of the working week from Monday to Wednesday (22, 35, 41, 43, 44). The authors note the peak of the injury rate on Monday and attribute the increased frequency to the changes to tasks performed after the weekend (22, 44). In our case, however, the end of the week was the riskiest in terms of injury, possibly due to increasing fatigue from the previous days and lower concentration on work.

August was the month with the highest injury rate (23.1%), while the lowest values of 0% were reached in July and December, probably due to the summer and Christmas holidays, when the number of workers at work is low. Thus, this study did not show the effect of the month of the year on the injury frequency. In their studies, Wilhelmson et al. (34) and Errico et al. (41) report that most occupational accidents and fatalities occur in May. Tsioras et al. (24) list October as the month with the highest number of injuries.

Jankovský et al. (35) state that the most dangerous profession was feller (28%), followed by forester (16.3%) and repairman (mechanic) (13.1%). This trend is also consistent with our study. In terms of the activity performed, in our case, the riskiest work has been confirmed in plantations and cultivation activities, especially pruning. We attribute this to working with chainsaws or sharp objects in challenging terrain. The relatively low number of occupational accidents recorded by the company can be attributed to the large share of mechanized harvesting, used primarily in the coniferous forests managed by the company. Mechanized harvesting systems protect the workers in the most dangerous situations that stem from semi-mechanized chainsaw logging, as reported by Albizu-Urionabarrenetxea et al. (18), Peters (45), Thelin (33), Laschi et al. (22), and Allman et al. (36).

According to Laschi et al. (22), contusion was the most common injury, accounting for 36% of all injuries, followed by superficial wounds with 23% and “Dislocation, sprain, strain” (16%). Similarly, Tsioras et al. (24) also found contusions (37.8%) to be the most common injury type, though they were followed by fractures (12.8%) in that study. In our case, superficial wounds were the most common injury type (34.6%), followed by dislocations and sprains (30.8%), which corresponds to Wilmsen et al. (25), who report superficial wounds as the most common injury type (33%).

According to the results, the lower extremities were the most frequently affected body parts (50%), especially the knees and ankles (affected frequently by sprains and sprains). Lower extremities were followed by upper extremities with 19.2% and head with 7.7% shares. Lilley et al. (16) and Albizu-Urionabarrenetxea et al. (18) also mention, similarly to our study, that the most frequently injured body parts were the lower extremities. Consistent with our results, the upper and lower extremities are frequently listed as the most injured body parts, with Tsioras et al. (24) reporting 64%, Laschi et al. (22) reporting 65%, and Potočnik et al. (46) reporting 66%. For fatal accidents, it is the head that is mentioned as the most frequently affected body part (36, 41).

Consistent with this and other studies, materials and loads are the most common material agents in forestry. Most often, falling trees or their parts (tops or branches) cause accidents—Albizu-Urionabarrenetxea et al. (18) report 43% of injuries result from falling objects, whereas Rodriguez-Acosta and Loomis (47) attribute almost three-quarters of injuries (73.6%) to falling objects. On the other hand, Laschi et al. (22) report falling objects only as second in line, causing 14% of injuries, following forest ground with a 27% share on the total number of injuries. We came to similar results in our study, where workspaces and forest ground were the sources of falls (38.5%), followed by materials and loads (30.8%). We attribute this to the fact that we do not have suppliers for work performing harvesting activities.

## 5 Conclusion

We can conclude that the employee accident rate at MFE is somewhat lower than those reported by other authors dealing with forestry occupational accidents. As we already mentioned, the lower accident rate was mainly due to the suppliers carrying out the logging operations. Suppliers were obliged to report only severe and fatal occupational accidents to the company; other (minor) and registered accidents were not reported. From our point of view, it would be appropriate for suppliers to report all accidents that cause incapacity to work, primarily to obtain data and a detailed overview, thus enabling improvements to the OSH management system at the company. Despite this, no severe or fatal occupational accidents were reported by suppliers during the reporting period.

Based on the analyses we carried out, we can summarize the following recommendations, which should be relayed to employees during OSH training:

- Increase the number of training sessions for employees with a short period of experience below 5 years.
- Increase the frequency of training for employees over 60 years of age.
- Notify employees to maintain or increase their vigilance approximately 1–2 h before and after lunch.
- Notify employees of the need to increase concentration at the beginning and end of the work week.
- Increase the frequency of safety training, especially for employees working in the field.
- Ensure strict adherence to wearing good quality personal protective equipment, especially equipment that protects against falling objects.
- Incorporate results from studies on accidents in forestry in safety training, so that workers know the most frequent modes, material agents, time of occurrence, and activities during which occupational accidents occur. Exposure to this information can increase worker vigilance while performing the most dangerous tasks.

## Data availability statement

The raw data supporting the conclusions of this article will be made available by the authors, without undue reservation.

## Ethics statement

Ethical approval was not required for the study involving humans in accordance with the local legislation and institutional requirements. Written informed consent to participate in this study was not required from the participants or the participants’ legal guardians/next of kin in accordance with the national legislation and the institutional requirements.

## Author contributions

MA: Conceptualization, Data curation, Formal analysis, Investigation, Methodology, Supervision, Validation, Visualization, Writing – original draft, Writing – review & editing. ZD: Conceptualization, Data curation, Formal analysis, Investigation, Methodology, Resources, Validation, Visualization, Writing – original draft, Writing – review & editing. MJ: Conceptualization, Formal analysis, Methodology, Project administration, Validation, Visualization, Writing – original draft, Writing – review & editing.

## References

[1] FAO (2022). The State of the World’s Forests 2022. Forest Pathways for Green Recovery and Building Inclusive, Resilient and Sustainable Economies. Rome, Italy: Food and Agriculture organization. Forest pathways for green recovery and building inclusive, resilient and sustainable economies.

[2] Nadrowski K Wirth C Scherer-Lorenzen M. Is Forest diversity driving ecosystem function and service? Curr Opin Environ Sustain. (2010) 2:75–9. doi: 10.1016/j.cosust.2010.02.003

[3] Pilli R Pase A. Forest functions and space: a Geohistorical perspective of European forests. IForest Biogeosci Forest. (2018) 11:79–89. doi: 10.3832/ifor2316-010

[4] Hruza P Vyskot I. Social-recreation evaluation of Forest roads and their suitability for trails: towards a complex approach. Croat J Forest Eng. (2010) 31:127–35.

[5] Wilkes-Allemann J Hanewinkel M Pütz M. Forest recreation as a governance problem: four case studies from Switzerland. Eur J For Res. (2017) 136:511–26. doi: 10.1007/s10342-017-1049-0

[6] Kindler E. A comparison of the concepts: ecosystem services and Forest functions to improve interdisciplinary exchange. Forest Policy Econ. (2016) 67:52–9. doi: 10.1016/j.forpol.2016.03.011

[7] Zhang Q Li Y Chang Y Qi J Yang C Cheng B. Global timber harvest footprints of nations and virtual timber trade flows. J Clean Prod. (2020) 250:119503. doi: 10.1016/j.jclepro.2019.119503

[8] Cabral JP Kafle B Subhani M Reiner J Ashraf M. Densification of timber: a review on the process, material properties, and application. J Wood Sci. (2022) 68:20. doi: 10.1186/s10086-022-02028-3

[9] Ramage MH Burridge H Busse-Wicher M Fereday G Reynolds T Shah DU. The wood from the trees: the use of timber in construction. Renew Sust Energ Rev. (2017) 68:333–59. doi: 10.1016/j.rser.2016.09.107

[10] FAO, ILO, and United Nations (2023). Occupational safety and health in the future of forestry work. Forestry working paper 37. Rome, Italy: FAO, ILO, United Nations. Available at: https://www.fao.org/3/cc6723en/cc6723en.pdf

[11] Ackerknecht C. Work in the forestry sector: some issues for a changing workforce. Unasylva. (2010) 61:60–6.

[12] ILO (2019). “Conclusions on promoting decent work and safety and health in forestry. SMSHF/2019/9” in *Sectoral Meeting on Promoting Decent Work and Safety and Health in Forestry*. Geneva, Switzerland: International Labour Organization.

[13] Batista AG Santana VS Ferrite S. The recording of fatal work-related injuries in information Systems in Brazil. Ciênc Saúde Colet. (2019) 24:693–704. doi: 10.1590/1413-81232018243.35132016, PMID: 30892492

[14] Ceylan H. Analysis of occupational accidents according to the sectors in Turkey. Gazi Univ J Sci. (2012) 25:909–18.

[15] Klun J Medved M. Fatal accidents in forestry in some European countries. Croat J Forest Eng. (2007) 28:55–62.

[16] Lilley R Feyer AM Kirk P Gander P. A survey of Forest Workers in New Zealand: do hours of work, rest, and recovery play a role in accidents and injury? J Saf Res. (2002) 33:53–71. doi: 10.1016/S0022-4375(02)00003-8, PMID: 11979637

[17] Feyer A-M Williamson AM Stout N Driscoll T Usher H Langley JD. Comparison of work related fatal injuries in the United States, Australia, and New Zealand: method and overall findings. Inj Prev. (2001) 7:22–8. doi: 10.1136/ip.7.1.22, PMID: 11289530 PMC1730691

[18] Albizu-Urionabarrenetxea PM Tolosana-Esteban E Roman-Jordan E. Safety and health in Forest harvesting operations. Diagnosis and preventive actions. A review. Forest Syst. (2013) 22:392–400. doi: 10.5424/fs/2013223-02714

[19] Wiatrowski W Janocha J. Comparing fatal work injuries in the United States and the European Union. Monthly Labor Rev. (2014) 137. doi: 10.21916/mlr.2014.23

[20] Eurostat (2023). “Eurostat Statistics.” Available at: https://ec.europa.eu/eurostat/databrowser/explore/all/all_themes

[21] Cho MJ Choi YS Lee E. Identifying risk factors and evaluating occupational safety in south Korean forestry sector. Forests. (2023) 14:851. doi: 10.3390/f14040851

[22] Laschi A Marchi E Foderi C Neri F. Identifying causes, dynamics and consequences of work accidents in Forest operations in an alpine context. Saf Sci. (2016) 89:28–35. doi: 10.1016/j.ssci.2016.05.017

[23] Salminen S Klen T Ojanen K. Risk taking and accident frequency among Finnish forestry workers. Saf Sci. (1999) 33:143–53. doi: 10.1016/S0925-7535(99)00029-6

[24] Tsioras P Rottensteiner C Stampfer K. Analysis of accidents during cable yarding operations in Austria 1998–2008. Croat J Forest Eng. (2011) 32:549–560

[25] Wilmsen C Bush D Barton-Antonio D. Working in the shadows: safety and health in forestry Services in Southern Oregon. J For. (2015) 113:315–24. doi: 10.5849/jof.13-076, PMID: 29643572 PMC5890815

[26] Moravčík M Kovalčík M Kunca A Schwarz M Longauerová V Pajtík J. (2021). “Green report.” Bratislava, Slovakia: Ministry of Agriculture and Rural Development of the Slovac Republic, National Forest Center. Available at: file:///C:/users/dudakovaz/downloads/zelena_sprava_2021_skratena_verzia_pdf.

[27] Ministry of Labour, Social Affairs and Family of the Slovak Republic (2006). Regulation on the registered occupational accident template. Available at: https://www.slov-lex.sk/pravne-predpisy/SK/ZZ/2006/500/

[28] European Parliament and Council (1989). OSH “framework directive” of 12 June 1989 on the introduction of measures to encourage improvements in the safety and health of Workers at Work-“framework directive”.

[29] European Union. European Statistics on Accidents at Work (ESAW). Luxembourg: Publications Office of the European Union (2013).

[30] Akay AO Akgul M Esin Aİ Senturk N. Evaluation of occupational accidents in forestry in terms of incidence, frequency, and severity rates in Turkey. Int J For Eng. (2023) 34:26–34. doi: 10.1080/14942119.2022.2061889

[31] Peirano C. Addressing the safety of forest workers. Unasylva. (2012) 63:7.

[32] Grzywiński W Turowski R Naskrent B Jelonek T Tomczak A. The effect of season of the year on the frequency and degree of damage during commercial thinning in black Alder stands in Poland. Forests. (2019) 10:668. doi: 10.3390/f10080668

[33] Thelin A. Fatal accidents in Swedish farming and forestry, 1988–1997. Saf Sci. (2002) 40:501–17. doi: 10.1016/S0925-7535(01)00017-0

[34] Wilhelmson E Wästerlund DS Burström L Bylund PO. Public health effects of accidents in self-employed forestry work. Small Scale For Econ Manag Policy. (2005) 4:427–35. doi: 10.1007/s11842-005-0026-5

[35] Jankovský M Allman M Allmanová Z. What are the occupational risks in forestry? Results of a long-term study in Slovakia. Int J Environ Res Public Health. (2019) 16:4931. doi: 10.3390/ijerph16244931, PMID: 31817497 PMC6949895

[36] Allman M Dudáková Z Jankovský M. Long-term temporal analysis of fatal and severe occupational accidents in central European forests of the Slovak Republic. J Saf Res. (2023) 87:488–95. doi: 10.1016/j.jsr.2023.09.002, PMID: 38081720

[37] Bentley TA Parker RJ Ashby L. Understanding felling safety in the new Zealand Forest industry. Appl Ergon. (2005) 36:165–75. doi: 10.1016/j.apergo.2004.10.009, PMID: 15694070

[38] Bentley TA Parker RJ Ashby L Moore DJ Tappin DC. The role of the new Zealand Forest industry injury surveillance system in a strategic ergonomics, safety and Health Research Programme. Appl Ergon. (2002) 33:395–403. doi: 10.1016/S0003-6870(02)00037-6, PMID: 12236648

[39] Lefort AJ de Hoop CF Pine JC Marx BD. Characteristics of injuries in the logging industry of Louisiana, USA: 1986 to 1998. Int J For Eng. (2003) 14:75–89. doi: 10.1080/14942119.2003.10702480

[40] Wang X Yuanchang L Xing H Zeng J Xie Y Cai D. Effects of close-to-nature conversion on *Pinus Massoniana* plantations at different stand developmental stages. Trop Conserv Sci. (2018) 11:194008291876795–16. doi: 10.1177/1940082918767953

[41] Errico S Drommi M Calamano V Barranco R Molinari G Ventura F. Fatal work-related injuries in the Genoa District (North-Western Italy): forensic analysis of the 10-year period between 2011 and 2020. J Forensic Leg Med. (2022) 85:102294. doi: 10.1016/j.jflm.2021.102294, PMID: 34864389

[42] Driscoll TR Ansari G Harrison JE Frommer MS Ruck EA. Traumatic work-related fatalities in forestry and sawmill Workers in Australia. J Saf Res. (1995) 26:221–33. doi: 10.1016/0022-4375(95)00018-L

[43] Pickett W Hartling L Brison RJ Guernsey JR. Fatal work-related farm injuries in Canada, 1991–1995. Can Med Assoc J. (1999) 160:1843–8. PMID: 10405669 PMC1230438

[44] Tsioras PA Rottensteiner C Stampfer K. Wood harvesting accidents in the Austrian state Forest Enterprise 2000–2009. Saf Sci. (2014) 62:400–8. doi: 10.1016/j.ssci.2013.09.016

[45] Peters PA. Chainsaw felling fatal accidents. Transact ASAE. (1991) 34:2600–8. doi: 10.13031/2013.31912

[46] Potočnik I Pentek T Poje A. Severity analysis of accidents in forest operations. Croatian Journal of Forest Engineering: Journal for Theory and Application of Forestry Engineering. (2009) 30:171–184.

[47] Rodriguez-Acosta RL Loomis DP. Fatal occupational injuries in the forestry and logging industry in North Carolina, 1977–1991. Int J Occup Environ Health. (1997) 3:259–65. doi: 10.1179/oeh.1997.3.4.259 9891126

